# Repatriation, identity, and organisational adaptation: a case study on reverse brain drain and socio-psychological drivers in Turkey’s energy sector

**DOI:** 10.3389/fpsyg.2026.1802059

**Published:** 2026-10-02

**Authors:** Cihan Durmuşkaya, Mustafa Abdül Metin Dinçer, Onur Metin, Akın Özdemir

**Affiliations:** 1 Department of Labour Economics and Industrial Relations, Faculty of Political Sciences, Sakarya University, Sakarya, Türkiye; 2 Human Resources Management Program, Department of Management and Organisation, Geyve Vocational School, Sakarya University of Applied Sciences, Sakarya, Türkiye; 3 Sakarya Economic and Social Research Center (SESAM), Sakarya University, Sakarya, Türkiye

**Keywords:** institutional logics, knowledge transfer, organisational socialisation, qualitative case study, reality shock, return migration, scholarship-bound mobility

## Abstract

This study examines the post-return experiences of engineers who completed postgraduate education abroad under a state scholarship carrying a service-or-repay bond and returned to a strategic public organisation in Turkey, the Case Company (CC): graduates either serve the institution for twice the funded period or reimburse the full cost of their training. We characterise this arrangement as scholarship-bound return migration and direct attention to what the bond cannot secure: integration after return. The research focuses on their integration into local work processes, organisational adaptation dynamics, knowledge transfer mechanisms, and institutional logic conflicts. In this context, the research question of the study has been formulated as follows: How do the socio-psychological experiences of scholarship-funded engineers evolve after their return, as they adapt to their home organisation and its competing institutional logics? Using a qualitative methodology, we conducted semi-structured interviews with 12 engineers who returned under this scholarship-bound arrangement and analysed the data using inductive and deductive content analysis in MAXQDA. Findings indicate that participants experienced reality shock due to inadequate orientation, alongside professional dissatisfaction stemming from expertise-role mismatches. However, socio-cultural factors—specifically a ‘debt of gratitude’ and strong family ties—prove pivotal in retention. Analysis through an institutional logics lens revealed persistent tensions between CC’s public-bureaucratic operating logic and the global market logic that engineers had internalised during their studies abroad. While existing literature focuses on macro-level migration factors, this study addresses micro-level organisational adaptation and cognitive dissonance within strategic sectors through institutional logic analysis.

## 1 Introduction

In recent years, reverse brain drain has emerged as a salient labour market policy in developing countries to repatriate skilled human capital and enhance national competitiveness. The return of internationally educated professionals offers potential for knowledge transfer, innovation and sectoral development (Docquier and Rapoport, 2012; Saxenian, 2005). However, their integration into post-return work processes and adaptation to local institutional structures remain poorly understood—dynamics that shape individual careers, national innovation policies, and sectoral strategies, particularly in strategic sectors like oil and gas (Kwok and Leland, 1982; Dustmann et al., 2011).

Academic studies on reverse brain drain in Turkey have predominantly focused on macro-level migration causes and policy consequences (Güngör and Tansel, 2007, 2014; Köser-Akçapar, 2006; Yılmaz-Şener and Türgen, 2018). However, sectoral (meso) and institutional (micro) level studies remain limited. This study addresses this gap by examining engineers at CC, a critical public institution in Turkey’s oil and gas sector, exploring their integration into local work processes and institutional adaptation dynamics. In this context, the research question of this study is as follows: How do the socio-psychological experiences of scholarship-funded engineers evolve after their return, as they adapt to their home organisation and its competing institutional logics?

It should be noted that CC’s scholarship programme, administered by the Ministry of National Education, enables engineers to receive international training. Upon return, they integrate into field and office duties. However, limited information exists regarding their transition experiences and adaptation processes.

A note on terminology is needed here. The broader literature reserves reverse brain drain for voluntary, incentive-driven return flows and brain circulation for sustained multidirectional mobility (Saxenian, 2005; Zweig, 2006). The case examined here fits neither comfortably. CC’s scholarship operates as a bond of the kind used in Singapore, Malaysia, and Kazakhstan’s Bolashak programme (Bokayev et al., 2020): graduates either return and serve the institution for twice the funded period, with full salary and standing, or remain abroad and repay the full cost of their funded education. Return is therefore neither guaranteed nor freely chosen; it is the default arm of a priced choice. Some scholarship holders repay and stay, and some who serve leave once the bond expires. We describe this arrangement as scholarship-bound return migration and study its returning arm: engineers who chose service over repayment. A bond of this kind can compel presence; whether it produces integration is the question this study asks.

This research employs four theoretical approaches: organisational socialisation theory (Van Maanen and Schein, 1979), reverse brain drain (Zweig, 2006), knowledge transfer theory (Nonaka, 1994), and institutional logics (Friedland and Alford, 1991). Through thematic content analysis of semi-structured interviews using MAXQDA, we examine how returning engineers transfer global knowledge and navigate institutional conflicts. The study offers institution-level recommendations addressing orientation design, talent placement, and the development of academic-professional linkages within CC’s repatriation framework. Turkey’s oil and gas sector experiences may offer comparative insights for other developing countries’ reverse brain drain policies.

## 2 Conceptual and theoretical framework

### 2.1 Conceptual background

The term ‘reverse brain drain’ is commonly used for professionals who return to their countries of origin after receiving education or professional experience abroad to utilise their knowledge in local contexts; Lowell and Findlay (2002), for instance, discuss policy responses aimed at reversing brain drain without offering a formal definition of the term. In developing countries, this offers strategic recovery of qualified human capital and innovation potential. However, returnees’ integration is shaped by organisational dynamics, cultural factors, and conflicting logics (Thornton and Ocasio, 2008). The literature proposes various concepts: brain gain (knowledge contribution), brain circulation (talent mobility across networks), technology transfer (two-way mobility), brain drain (talent loss), and “brain dump” (reverse knowledge migration, Saxenian, 2002a). These related terms warrant distinction. Brain drain denotes the sustained loss of skilled human capital to destination countries; reverse brain drain describes the voluntary, large-scale return of that population; brain circulation captures ongoing, multidirectional mobility; and repatriation refers to the individual-level process of returning to and re-entering home institutions. The present case combines features of the latter two, with one structural difference: mobility here is organised by a service-or-repay bond rather than by market incentives or open circulation. Participants are repatriates who took the service arm of that bond, which is why we frame the phenomenon as scholarship-bound return migration and focus on the post-return period rather than the return decision.

Studies demonstrate both individual and structural factors influence this phenomenon. Individual factors include career expectations, economic opportunities, quality of life, cultural belonging, and family ties (Kwok and Leland, 1982; Tharenou and Seet, 2014). Structural factors encompass economic stability, science and technology infrastructure, and employment policies (Docquier and Rapoport, 2012; Chacko, 2007). The ‘push and pull factors’ concept emphasises external and internal constraints in reverse brain drain (Zweig, 2006; Sahay, 2014). The following table provides a concise overview of the extant studies in this field, organised according to their research areas, focuses, key findings and contributions.

As Table 1 illustrates, research on reverse brain drain has developed across nine thematic areas, each approaching the phenomenon from a distinct analytical vantage point. Studies focused on geographical distribution and diaspora networks establish the macro-structural conditions under which return migration becomes viable. At the same time, education and career-oriented research attends to the individual calculus behind mobility decisions. Economic and remittance-focused work situates reverse brain drain within broader development debates, and family and social factors research consistently demonstrates that non-economic ties—particularly cultural belonging and familial obligations—carry substantial explanatory weight. The remaining thematic areas, covering research infrastructure, economic stability, and social justice perceptions, collectively point to the multidimensionality of return decisions. Taken together, these strands suggest that reverse brain drain cannot be reduced to a single driver; it emerges from the intersection of structural conditions, institutional incentives, and deeply personal attachments. The present study contributes to this body of work by examining the micro-level organisational dynamics that follow the return decision—a dimension that remains comparatively underexplored across all nine thematic areas.

**Table 1:** Key dimensions and findings of studies in the literature on reverse brain drain.

**Thematic area / Dimension**	**Research focuses**	**Key findings / Contributions**	**Selected sources**
Geographical focus	Asia, Latin America, and the Pacific regions	It has been noted that reverse brain drain processes are intensively studied in these regions.	Zweig (2006), Nguyen et al. (2006), Gibson and McKenzie (2011), Astor et al. (2005)
Diaspora and knowledge networks	The contribution of diaspora networks to economic development, knowledge sharing and innovation	Supports economic development, facilitates knowledge sharing, and strengthens innovation and brain circulation.	Tung and Lazarova (2006), Giannetti et al. (2015), Bosetti et al. (2015), Hussain (2015), Beine et al. (2011)
Education dimension	The impact of education investments and opportunities on the decision to migrate	International education functions not only as financial investment but also as a pathway to self-realisation and cultural development; good education opportunities encourage both migration and return decisions.	Mountford (1997), Ha et al. (2016), Di Maria and Lazarova (2012), Koska et al. (2013), Williams (2007), Dulam and Franses (2015), Cebolla-Boado et al. (2017)
Career and remuneration	The role of career opportunities and income expectations	Salary and career opportunities are decisive factors in migration and reverse migration decisions.	Kwok and Leland (1982), Sjaastad (1962), Eggert et al. (2010), Bokayev et al. (2020), Artuç et al. (2015)
Research opportunities	The influence of scientific and academic environments	Research infrastructure and opportunities are a significant factor in the decision of qualified individuals to return to their home countries.	Jonkers and Tijssen (2008), Michaelis (1990), Vizi (1993)
Economic and financial impacts	The effects of remittances on economic growth and development	Remittances contribute to countries’ financial systems, but brain drain may slow growth in the long term.	Page and Plaza (2006), Taylor (1999), Bollard et al. (2011), Desai et al. (2009), Li et al. (2017)
Family and social factors	Family ties, cultural belonging and social support systems	Cultural comfort, familiar surroundings, and family ties function as primary pull factors in return decisions; social bonds consistently outweigh purely economic motivations.	Ifanti et al. (2014), Ibrahim et al. (2019), Poppe et al. (2014), Tharenou and Seet (2014)
Economic stability	The role of macroeconomic conditions in migration	Economic stability is a decisive factor in both migration and reverse migration decisions.	Okeke (2013), Bartolini et al. (2017)
Social justice and equality	Perception of equal opportunity and social justice	The perception of social justice and equality is a cultural factor that influences reverse migration motivation.	Facchini and Mayda (2009), Zweig et al. (2021)

As can be observed from Table 1, Cebolla-Boado et al. (2017) posit that international education serves not only as financial investment but also as self-realisation and cultural experience. Return decisions depend on how this contributes to self-perception and objectives. Tharenou and Seet (2014) identify cultural comfort, familiar surroundings, and loyalty as attractive factors, with family and friendship ties consistently cited as primary pull factors. State resources and government policies shape return decisions by creating competitive professional environments and structured incentive frameworks (Zweig, 2006; Gibson and McKenzie, 2011).

In South Korea, Taiwan, India, and China, policymakers have cultivated favourable environments through comprehensive policies, financial incentives, and support for skilled human capital (Zweig, 2006; Zweig et al., 2021). CC’s scholarship system operates on a comparable compensation model: engineers either reimburse their scholarship costs or fulfil compulsory service obligations upon return—a structure that simultaneously incentivises repatriation and shapes post-return institutional commitment.

### 2.2 Theoretical framework

The following perspectives have been identified as relevant to this study: the reverse brain drain literature, organisational socialisation theory, knowledge transfer theory, and the institutional logics approach.

Building on this foundation, the study turns to the reverse brain drain literature, in particular case-based work spanning China, India, Taiwan, and the Pacific (Zweig, 2006; Saxenian, 2005; Gibson and McKenzie, 2011). It examines not why engineers returned, which the scholarship bond largely settled, but how that pre-committed return shaped their institutional experience afterwards.

Organisational socialisation theory examines how newcomers come to understand and internalise the norms, roles, and expectations of their workplace (Van Maanen and Schein, 1979). A consistent finding across this literature is that the early period of organisational entry is marked by uncertainty: Louis (1980) describes this as a process of sense-making in which prior expectations collide with encountered realities. How this uncertainty resolves depends in part on the quality of socialisation content—Chao et al. (1994) demonstrate that richer socialisation experiences predict stronger performance outcomes—and on the frequency of social interaction within the organisation (Reichers, 1987). For professionals undergoing role transitions rather than initial entry, Moeller (2024) argues that resocialisation demands particular institutional attention, as prior professional identities complicate rather than facilitate adaptation. Within this study, this theoretical lens frames the engineers’ post-return experience as a resocialisation process—one in which prior professional identities are renegotiated against CC’s established institutional norms. The foundational statements of this theory date to the late 1970s, but its core mechanisms have held up under newer conditions: recent work extends the framework to digital environments (Srinivas and Vanka, 2022), non-standard organisational settings (Traeger et al., 2021), and, closest to our case, the resocialisation of professionals in role transition (Moeller, 2024). We read the classical framework through this updated lens.

Furthermore, knowledge transfer theory examines transferring abroad-acquired knowledge into local processes, supporting innovation and creativity (Nonaka, 1994). Cohen and Levinthal (1990) introduced ‘absorptive capacity,’ explaining how individuals and receiving organisations benefit from knowledge transfer. As this study examines how engineers transfer global knowledge to local processes, knowledge transfer theory provides a significant analytical framework.

Finally, the institutional logics approach explains how fundamental belief systems shape individual behaviour and organisational structures (Thornton and Ocasio, 2008; Friedland and Alford, 1991). This provides a suitable framework for elucidating contradictions between public and global education logics. We employ this approach to examine engineers’ institutional logics pre-emigration, how abroad experiences influenced these logics, and logic changes/conflicts post-employment. This approach enhances analysis depth by elucidating rationales underpinning engineers’ actions through alternative theoretical frameworks. Of these four perspectives, organisational socialisation theory and the institutional logics approach carry the main explanatory weight in this study: the former accounts for how engineers re-enter and renegotiate their roles, the latter for why that renegotiation proves so difficult. The reverse brain drain literature and knowledge transfer theory play a contextual part, situating the case and informing the coding frame rather than driving the interpretation.

## 3 Materials and methods

This study employs a case study research strategy and qualitative content analysis. Case study research effectively examines phenomena with uncertain boundaries (Yin, 2003; Patton, 2002), offering unique opportunities for understanding social, individual, political, organisational, and group phenomena.

Data were collected through semi-structured interviews from the participants who constituted the cases of the study. Participants were selected through purposive sampling based on three explicit inclusion criteria: (1) current employment at CC at the time of the interview, (2) completion of postgraduate education abroad through CC’s state-administered scholarship programme, and (3) active return to professional duties at CC following the completion of the scholarship period. Engineers who had not yet completed their compulsory service obligations or who had left CC prior to the interview were excluded. This selection strategy ensured that all participants had direct, first-hand experience of the scholarship-bound return and post-return adaptation processes under investigation. The interviews were conducted face-to-face and over the phone. The interviews with each participant lasted between 45 and 75 min. The dates, times, and locations of the interviews with the participants are provided in Supplementary Table 1. Each interview was transcribed into Word and analysed with MAXQDA. Instead of calculating a quantitative inter-coder reliability coefficient (e.g., Cohen’s Kappa coefficient) for the coding of data obtained from the interviews, the researchers followed the multi-stage data protocol (Credibility, Transferability, Dependability, Confirmability) proposed by Lincoln and Guba (1985). Within this framework, in the first stage, each researcher independently reviewed the data collected in the field to create a preliminary code list. While creating the preliminary code list, each researcher used Klafki’s (2001, p. 145) spiral reading technique. During this process, the researchers read the data multiple times, took notes, and created the preliminary code list. In the second stage, the researchers met to compare their independent codings and sought consensus on shared codes and categories. They carried out this process over three meetings, and based on the consensus reached regarding the code and category lists, the data were entered into MAXQDA for analysis by a single researcher. The authors drew on both deductive and inductive approaches to build codes and categories. For the deductive part, the codes and categories were derived from related literature such as organisational socialisation theory (Van Maanen and Schein, 1979; Bauer and Erdogan, 2011), the reverse brain drain literature (Saxenian, 2005; Zweig, 2006) and knowledge transfer theory (Nonaka, 1994). In the inductive part of the code and category-building process, researchers derived codes and categories from interviewees’ statements as open codes. The list of codes derived from the literature and fieldwork is presented in Table 2. In order to contribute to our knowledge about the phenomenon we investigate in our study, we used a single case study method with multiple sub-dimensions as a research strategy (Yin, 2003). In this framework, the researchers conducted interviews with multiple cases in a single organisation, depending on the uniqueness of this phenomenon, and the cases were selected from those who had studied in different countries on a state-funded study abroad scholarship. Information regarding CC’s institutional integration procedures was gathered through the semi-structured interview protocol itself. The protocol included questions specifically targeting participants’ experiences with orientation processes, systemic support structures, institutional expectations upon return, and the alignment between their acquired expertise and assigned roles. The full interview protocol is provided as Supplementary Data 1.

**Table 2:** List of inductive and deductive codes.

**Category**	**Deductive codes (literature-derived)**	**Inductive codes (data-derived / open coding)**
Labour relations	Vocational Training	—
	Competence Development	—
Relationship between employee’s psychology and work life	Commitment	*Knowledge and Practice Adaptation*
	Burnout/Desperation/ Lack of Appreciation	*New Beginnings and Attempts for the Future*
	Foresight and Job Satisfaction	*Unsatisfaction due to Some Problems*
	—	*Uncertainty about Career Future*
	—	*Specialisation and Incompatible Field Experience*
	—	*Reality Shock*
Academic context	Groups/Projects	*Collaboration Requirements with Academy*
	—	*Orientation Support for the Academic Choices*
	Research and Education Support/Academic Career Continuity	—
	—	*Academic Support Requirement*
	—	*Uncertainty About Academic Career*
	—	*Subsequent Adaptation to the Academic System*
	—	*Disorganisation and Non-Adaptation to Academic System*
	—	*System Awareness and Adaptation to Academic System*
Institutional, political and economic factors (Bureaucratic structure)	Privatisation Demands	*Structural, Bureaucratic and Hierarchical Slowness*
	Professionalism Requirements/Demands	*Orientation Support for the Newcomers*
	Market and Organisational Culture Incompatibility	*Need for Internship Programme*
	—	*Systemic Support Adjustment*
	—	*Need for a Detailed Orientation and Supervisorship Programme*
Family, social ties and economic hardship	Debt of Gratitude	*Too Embedded in the System to Leave*
	Family Ties	*Poor Economic Conditions*
	Social Bonds	—
Cultural enrichment	Cultural Enrichment	—
	Adaptation	—
Economic stability	Pandemic Impacts	—
	Sectoral Uncertainties	—
Scholarship system, compulsory service	Compensation Model ‘Implicit Obligation’	*Lack of Orientation*
	Poor Contact/Deficiencies	*Conscious Choice*
	—	*To Be Informed by Chance*
	—	*Hardship in Transition Processes*
	—	*Easy Transition to the System*
	—	*Fair Selection System*
Political stability and security	Safety Concerns	—
	Political Risks	—

Reverse brain drain literature served as a theoretical lens and an analytical tool for understanding how abroad-acquired knowledge integrated into work processes and organisational effects. The institutional logics approach (Friedland and Alford, 1991) provided analytical perspective on relationship mechanisms between categories.

## 4 Findings

### 4.1 Code and categories

In line with qualitative analysis conventions (Saldaña, 2021), coding functions as the analysis foundation, providing descriptive labels and initiating the analytical process (Punch, 2005). Codes are labels or descriptions applied to data groups, from single words to large word sets. Saldaña (2021) defines typical coding as summarising data essence with evocative labels. Researchers created codes and categories from study data and literature definitions. Key relationships between singularity and multiplicity constitute patterns (Saldaña, 2021), with code convergences forming categories, and category convergences forming themes.

Our analysis codes and categories were shaped by conceptual tools drawn from related literature. In the pre-analysis stage of the study, researchers defined one main theme, which is ‘Reverse Brain Drain,’ and nine categories, namely: “Labour Relations, Relationship Between Employee’s Psychology and Work Life, Academic Context, Institutional, Political and Economic Factors Connected Bureaucratic Structure, Family, Social Ties and Economic Hardship, Cultural Enrichment, Economic Stability, Scholarship System, Compulsory Service, Political Stability and Security.”

Categories created by the researchers for the study and the codes contained in these categories are given in Figure 1, with Simple Mind Mapping Software to provide a more holistic perspective in the reader’s mind. Categories and codes used in the study are explained under headings.

**Figure 1:**
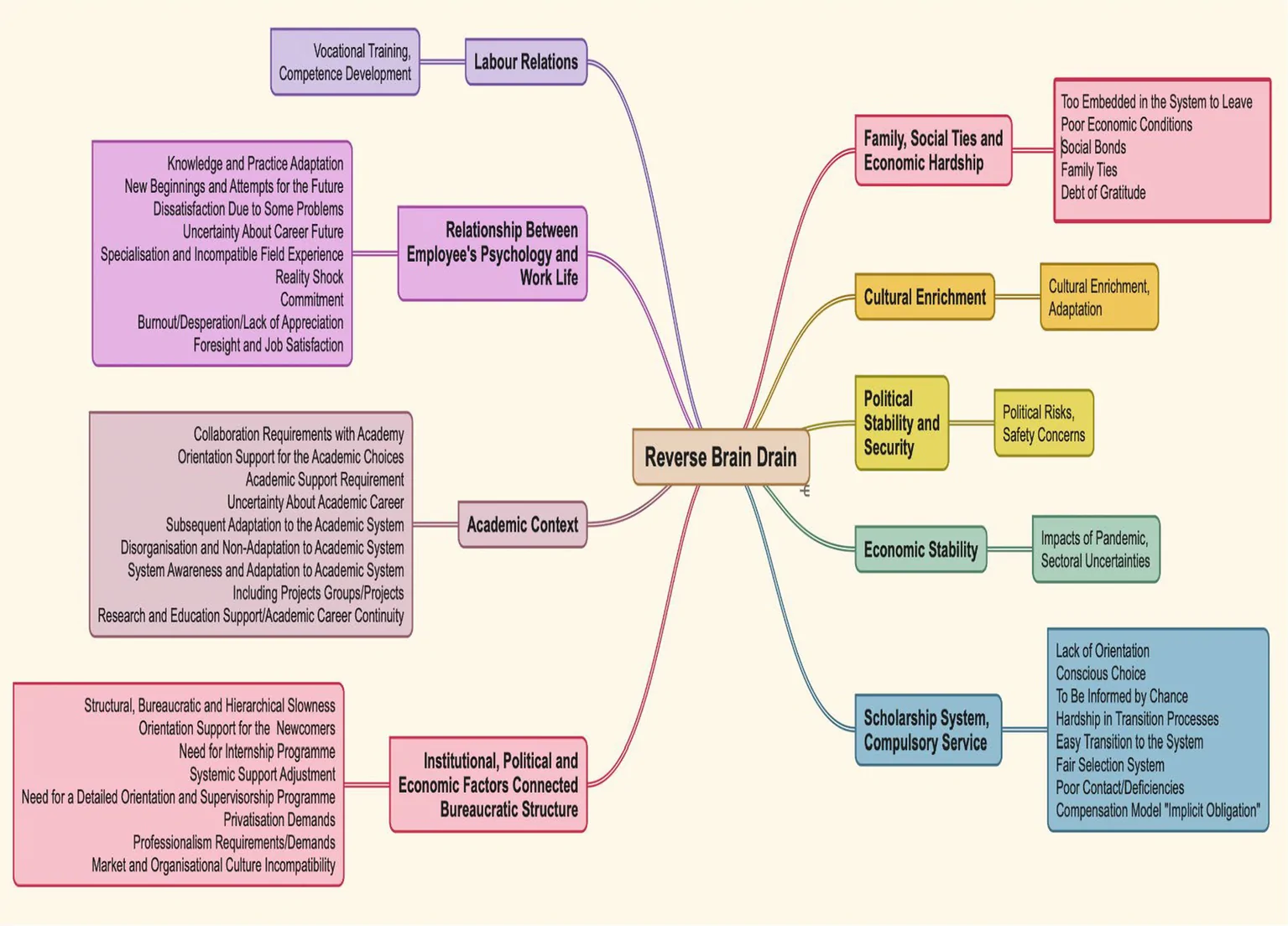
Mind map of codes and categories.

Below are the names referring to the studies behind the categories we produced within the framework of the data we mentioned in detail from the literature and the data we obtained from the field, and the explanations and codes for the categories:

The category of labour relations was created from the literature on the adaptation processes of individuals toward organisational norms and values based on organisational socialisation theory (Purnomo et al., 2022; Traeger et al., 2021; Van Maanen and Schein, 1979): Vocational Training, Competence Development.

The category of relationship between employee’s psychology and work life was created from the literature on employee adjustment, confusion, socialisation and intention to change jobs (Hoffmann and Thyregod Rasmussen, 2022; Louis, 1980): Commitment, Burnout/Desperation/Lack of Appreciation, Foresight and Job Satisfaction. Code created with open coding: Knowledge and Practice Adaptation, New Beginnings and Attempts for the Future, Unsatisfaction due to Some Problems, Uncertainty about Career Future, Specialisation and Incompatible Field Experience, Reality Shock.

The category of academic context was created from the relevant literature (Thornton and Ocasio, 2008; Argote and Ingram, 2000; Cohen and Levinthal, 1990; Friedland and Alford, 1991) to analyse the conflicting logics and processes between market mechanisms and other fields over time of the academic background acquired by employees; Including Projects Groups/Projects, Research and Education Support/Academic Career Continuity. Code created with open coding: Collaboration requirements with Academy, Orientation Support for the Academic Choices, Academic Support Requirement, Uncertainty About Academic Career, Subsequent Adaptation to the Academic System, Disorganisation and Non-Adaptation to Academic System, System Awareness and Adaptation to Academic System.

The category of institutional, political and economic factors connected bureaucratic structure was created based on the institutional logics, host and guest country and workforce literature (Thornton and Ocasio, 2008; Friedland and Alford, 1991; Tharenou and Seet, 2014; Jonkers and Cruz-Castro, 2013; Meyer and Rowan, 1977) in order to clarify the contradictions between CC’s public logics and global education logics and to analyse the contradictions within the framework of institutional logics; Privatisation Demands, Professionalism Requirements/Demands, Market and Organisational Culture Incompatibility. Code created with open coding: Structural, Bureaucratic and Hierarchical Slowness, Orientation Support for the Newcomers, Need for Internship programme, Systemic Support Adjustment, Need for a Detailed Orientation and Supervisorship Programme.

The category of Family, Social Ties and Economic Hardship is based on the relevant literature to analyse the relationships and dilemmas between migration motivations, economic stability, family ties, and career opportunities (Gibson and McKenzie, 2011; Astor et al., 2005); Debt of Gratitude, Family Ties, Social Bonds. Code created with open coding: Too Embedded in the System to Leave, Poor Economic Conditions.

The category of cultural enrichment was created from the relevant literature for the analysis of experiences gained at the point of cultural experience and experience (Cebolla-Boado et al., 2017; Williams, 2007); Cultural Enrichment, Adaptation.

The category of economic stability was created based on the relevant literature to examine the effect of economic stability and family ties in reverse migration (Güngör and Tansel, 2007; Tharenou and Seet, 2014); Pandemic Impacts, Sectoral Uncertainties.

The category of scholarship system and compulsory service was created from the literature on the effects of state resources and government policies on repatriation (Zweig, 2006; Kwok and Leland, 1982); Compensation Model “Implicit Obligation,” Poor Contact/Deficiencies. Code created with open coding: Lack of Orientation, Conscious Choice, To Be Informed by Chance, Hardship in Transition Processes, Easy Transition to the System, Fair Selection System.

The category of political stability and security was created based on the relevant literature to examine the effect of politics on the participants (Thornton and Ocasio, 2008; Friedland and Alford, 1991; Tharenou and Seet, 2014; Jonkers and Cruz-Castro, 2013; Meyer and Rowan, 1977): Safety Concerns, Political Risks.

### 4.2 General view of the findings

As illustrated in Figure 2, the MAXQDA matrix table reveals the overall view of the content analysis. The study’s cases are in the columns of the matrix table, while the study’s categories and codes are in the rows. The larger colourful boxes in Figure 2 show how dense the cases’ assertions are. Figure 2 suggests that the codes have literal replication—a pattern in which the same finding recurs consistently across multiple cases, lending cross-case validity to the identified themes (Yin, 2003). Across almost all categories, and for most participants, literal replication appears in these codes: “Foresight and Job Satisfaction, Specialisation and Incompatible Field Experience, Reality Shock,” “Compensation Model “Implicit Obligation,” Lack of Orientation, Conscious Choice,” “System Awareness and Adaptation to Academic System,” “Competence Development,” “Family Ties and Debt of Gratitude,” “Systemic Support Adjustment, Need for a Detailed Orientation and Supervisorship Programme.”

**Figure 2:**
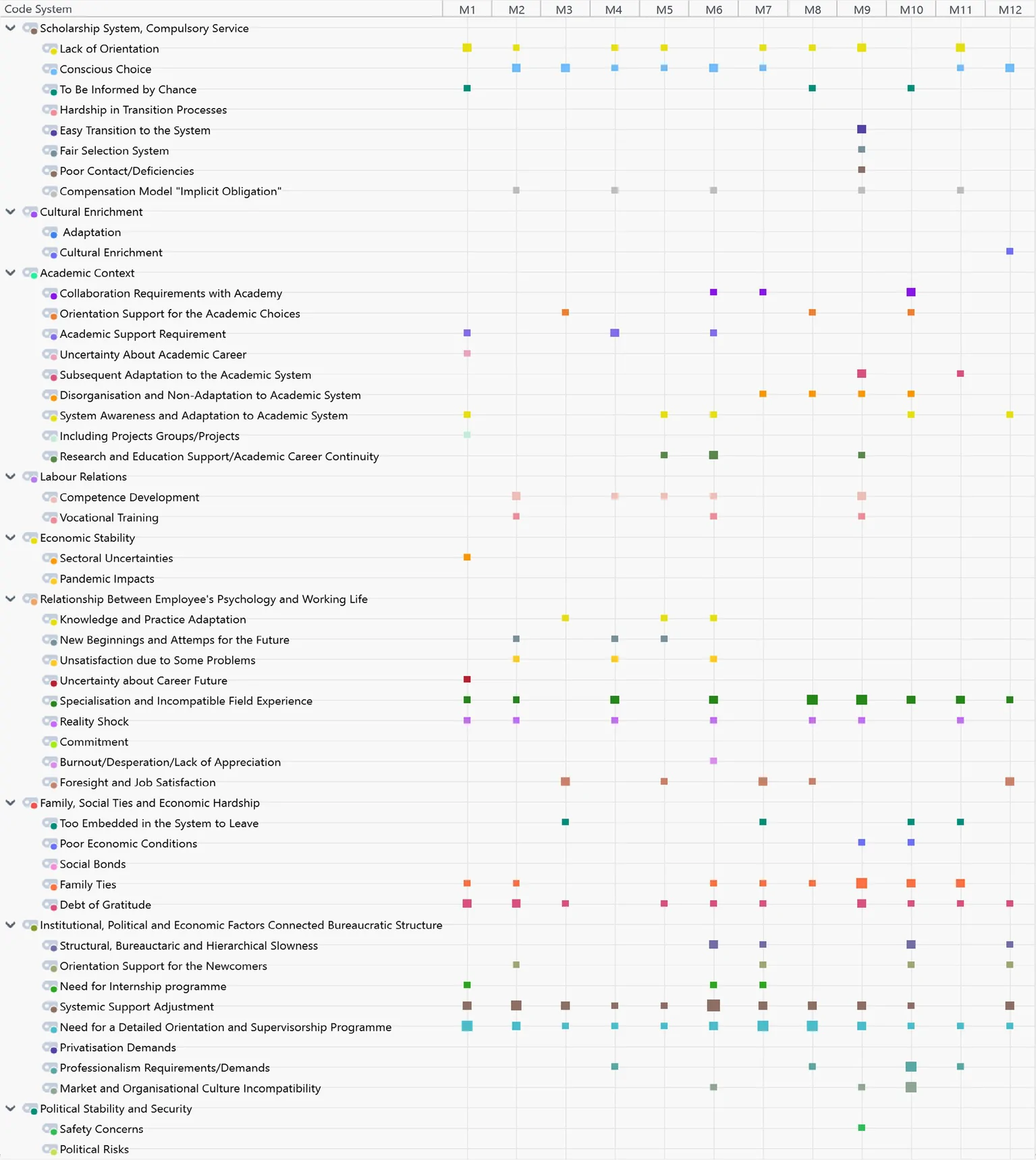
General view of the code matrix dispersion. This matrix table, generated via MAXQDA, visualizes the density of assertions across all 12 participants (M1–M12) relative to the identified research codes and categories.

Results are explained in detail below, concluding with a comprehensive graphic map.

## 5 Results and analysis

According to Figure 3, in this category “Conscious Choice” and “Lack of Orientation” codes have a significant dominance among others and they have the highest rank. The third code in this category is Compensation Model ‘Implicit Obligation’. In this category, it is seen that there is a conscious choice preference among the cases regarding scholarship application and a pattern of literal replication is observed among almost all cases. The second important issue in this category is the ‘Lack of Orientation’ code. Although the cases made a conscious choice, it is observed that the students who went abroad were not subjected to an orientation in terms of the processes, only an information meeting was held for the company in general, and in this case, the participants encountered various problems over time. In general, the participants stated that if an orientation for the process outside the company was given, they could resolve many of these difficulties without further complication, which reveals the necessity of a holistic approach not only at the company level but also at the scholarship process level. In the following categories, patterns related to orientation, especially within the company framework, were frequently observed and found an important place in the analysis in this framework. Some sample statements from cases:

**Figure 3:**
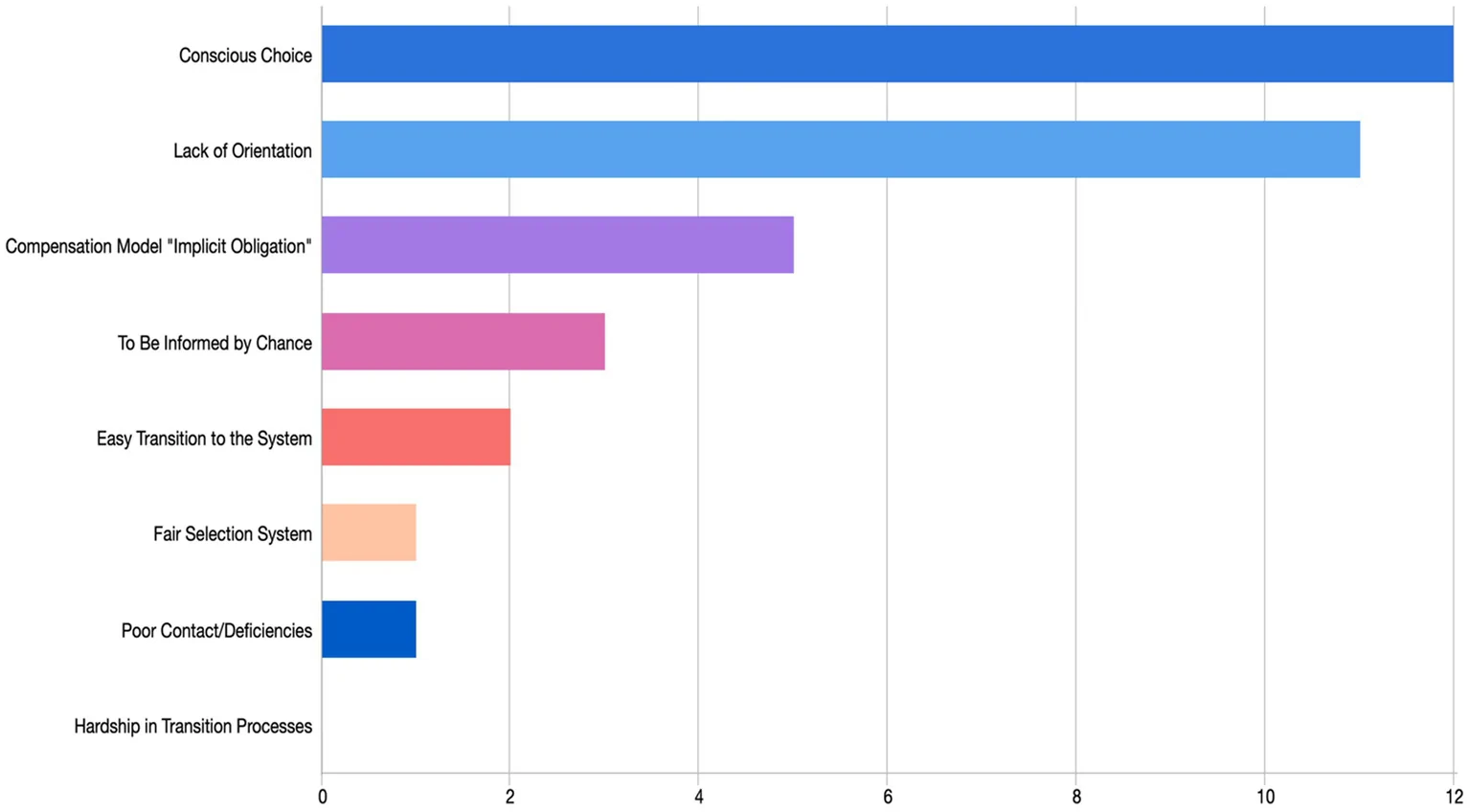
Category of scholarship system and compulsory service. This bar chart highlights the dominance of ‘‘Conscious Choice’’ and ‘‘Lack of Orientation’’ as the most frequent codes within the scholarship and compulsory service dimension.

“No information was provided before going abroad, only a general meeting. We found out process details ourselves.” — M4.

“After winning the scholarship, they held an introductory meeting there, but no one explained how the process would work. Then everyone encountered different problems.” — M11.

“I knew CC’s conditions were good, but without repayment obligation, I might have chosen another company.” — M6.

Two categories yielded little material. Cultural Enrichment drew statements from a single participant, as did Economic Stability, where only one engineer raised sectoral uncertainty. Both categories are documented in Supplementary Figures 1 and 2, but carry no interpretive weight in the analysis that follows.

According to Figure 4, the most frequently occurring code in this category is System Awareness and Adaptation to Academic System. It was observed that participants had a certain level of awareness about the functioning, rules, and processes of the academic system when they applied for the scholarship, but with the exception of one or two participants, none of them were able to continue their academic life after completing their master’s degree and then their doctorate. This situation appears to be a general pattern among participants and reinforces literal replication. Again, among the participants, the following four codes: Research and Education Support/Academic Career Continuity, Academic Support Requirement, Collaboration Requirements with Academy, Disorganisation and Non-Adaptation to Academic System, are in second place and show an equal distribution. The fundamental reason for this situation is the absence of a comprehensive relationship between the participants’ universities and companies, as well as the lack of collaboration with foreign universities and professors outside of scholarships. As a reflection of this situation, issues such as support for academic research and sustainability, and requests for support for success appear as a recurring pattern among the cases. Some sample statements from cases:

**Figure 4:**
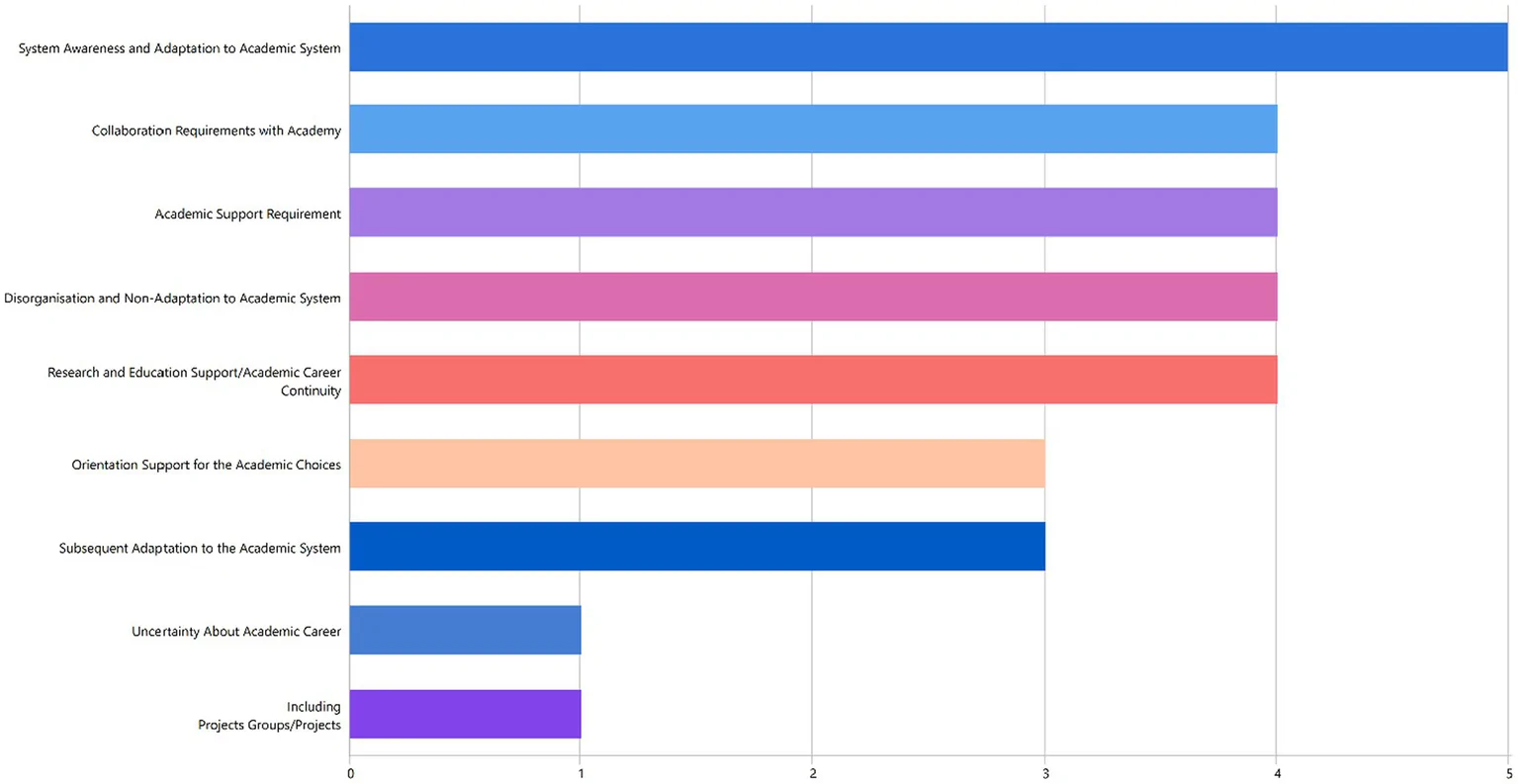
Category of academic context. This chart identifies ‘‘System Awareness and Adaptation to Academic System’’ as the most occurring code, reflecting the challenges returnees face in continuing academic pursuits post-employment.

“We contacted lecturers abroad and CC advisors, but upon return, those connections completely severed.” — M10.

“Every student has an advisor from the institution, but the process was not left to chance. The areas of work were determined according to CC’s needs.” — M3.

“If coordinated planning occurred with the institution during thesis periods, both we and the institution would have benefited.” — M8.

As illustrated in Figure 5, in this category, the “Competence Development” code shows significant prominence. The underlying reason is that participants were assigned to work in fields other than their areas of specialisation—areas they had mastered through graduate studies abroad—and therefore had to add new competencies to their expertise in the process. Parallel to this situation, the “Vocational Training” code also comes to the fore, indicating that the situation is a kind of training in a vocational sense. Some sample statements from cases:

**Figure 5:**
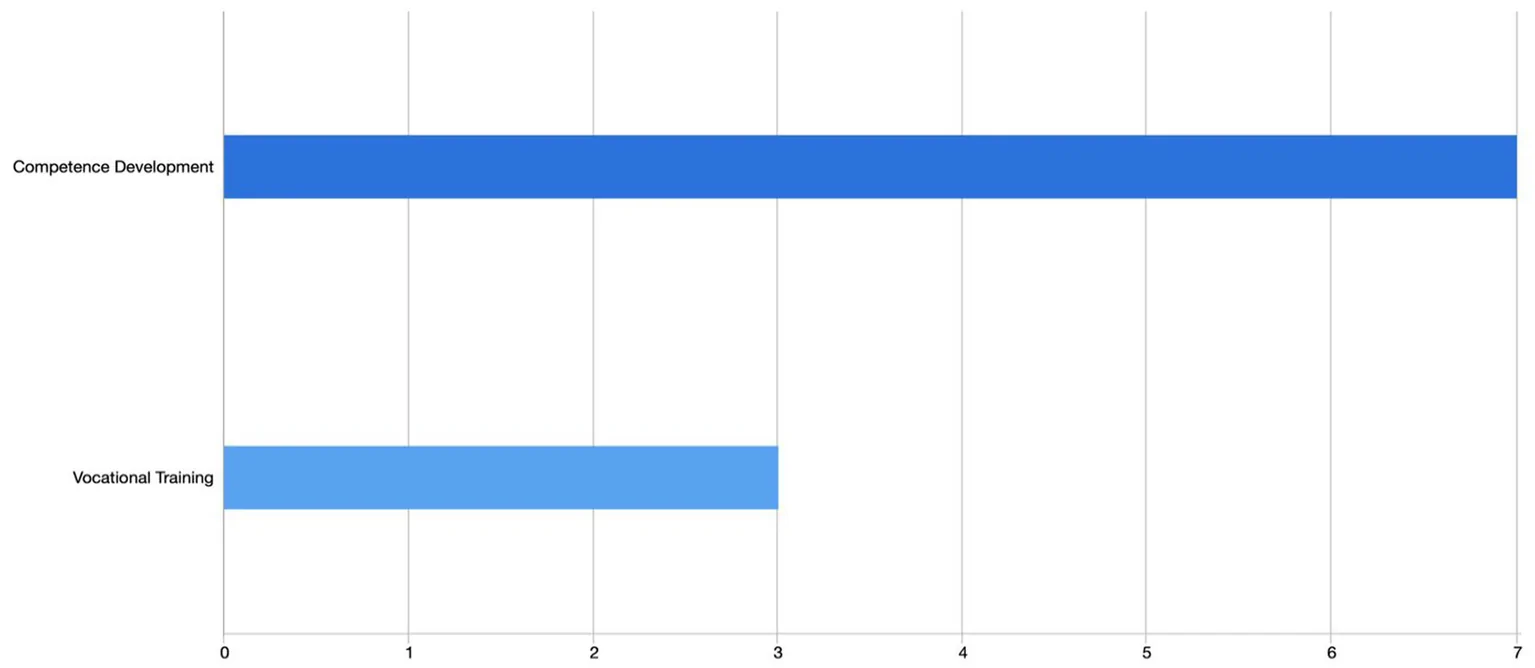
Category of labour relations. This figure illustrates the prominence of ‘‘Competence Development’’ and ‘‘Vocational Training,’’ arising from engineers being required to work in fields outside their primary specialization.

“Collaboration between universities and companies abroad was very strong. If we could create that environment here, we’d see scholarship benefits much more.” — M10.

“After winning the scholarship, they left everything to us. Acceptance and school selection were entirely down to personal effort.” — M6.

“Scholarship recipients really need to be selected from people who will actually do the job; otherwise, both the institution and the individual lose out.” — M7.

Figure 6 outlines that the most notable code in this category is “Specialisation and Incompatible Field Experience.” Most participants frequently mentioned the incompatibility between their specialisations and the work they did upon returning. This situation stands out as a negative issue in terms of professional satisfaction among participants. In contrast to this situation, the second most prominent code is “Foresight and Job Satisfaction.” Although these two situations appear to be contradictory, some participants were prepared for this situation thanks to the relationships they established with previous scholarship recipients. Another code that completes and reinforces this pattern is the “Reality Shock” code. After graduating from graduate school and returning to Turkey, the transition to the workforce, coupled with the expectations formed based on their overseas experiences, resulted in a reality shock, indicating that participants experienced a relatively short period of adjustment. Some sample statements from cases:

**Figure 6:**
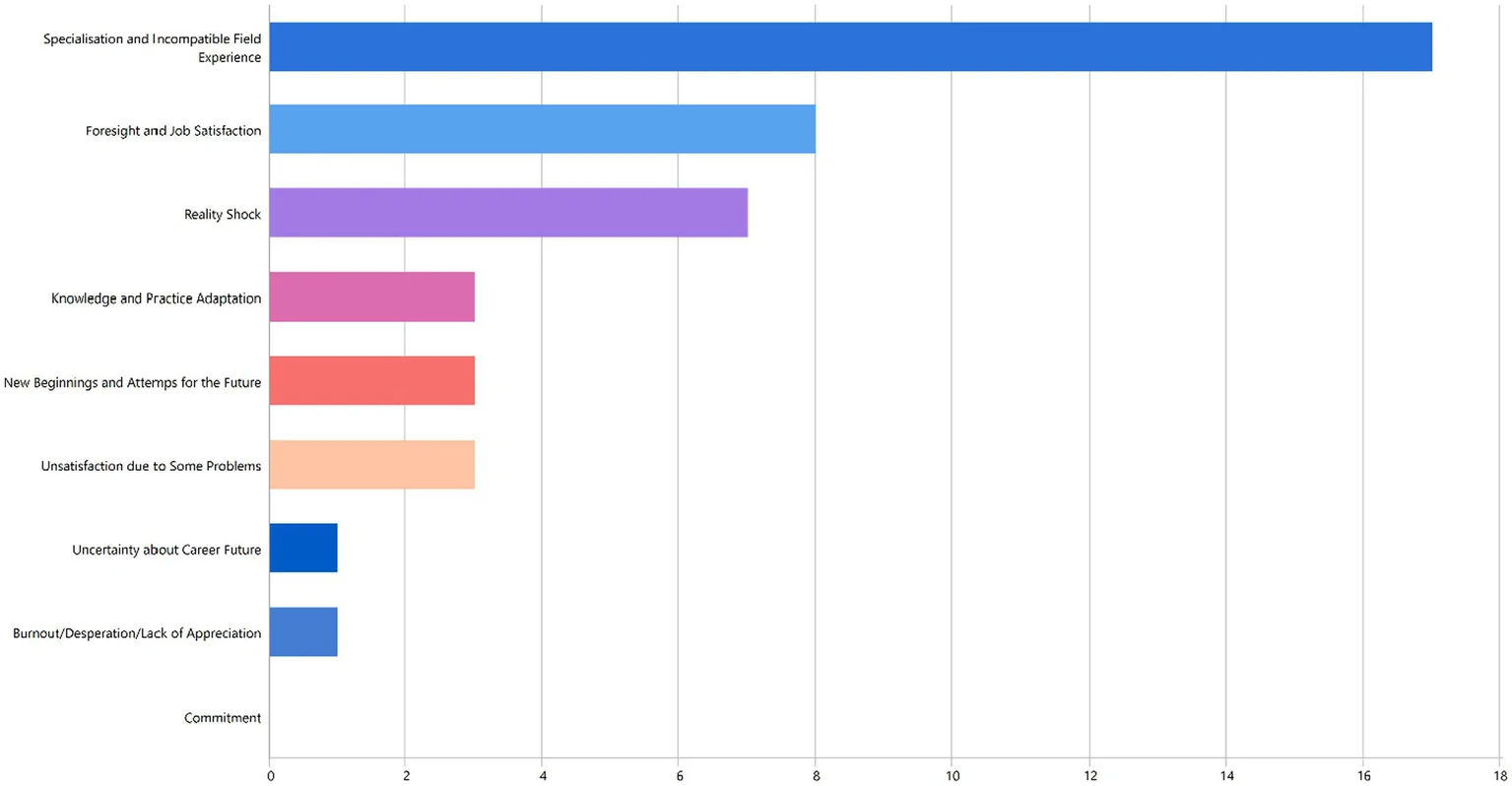
Category of Relationship between Psychology and Work Life. This bar chart identifies ‘‘Specialisation and Incompatible Field Experience’’ as a significant factor influencing professional dissatisfaction and the experience of ‘‘Reality Shock.’’

“After a while, metal fatigue sets in. Seeing success unappreciated wears you down.” — M6.

“I integrated my education into my work, but even though economic conditions are tough, I love my organisation and want to stay here.” — M12.

“I could not intern in the US, but had good ideas about Turkey work, so did not experience huge shock.” — M3.

As can be seen from Figure 7, the code with the highest rate in this category is “Debt of Gratitude,” which ranks first. The codes “Family Ties” and “Too Embedded in the System to Leave” share second place. An important point to note here is the relationship between “Debt of Gratitude” and the “Compensation Model ‘Implicit Obligation’” code. Although participants primarily express the “Debt of Gratitude” code, they also mention the “Compensation Model ‘Implicit Obligation’” code as an important underlying factor. This situation can be said to form the background for participants remaining strongly within the system under the influence of both codes. In second place, the “Family Ties” code stands out. Most participants stated that their families were also an important factor in their return. Another important code that completes the similarity of patterns between cases in this category and enables literal replication is the “Too Embedded in the System to Leave” code. Almost all participants who have completed a certain period of time at their institutions are now reluctant to leave for another country, citing both emotional and practical reasons. Some sample statements from cases:

**Figure 7:**
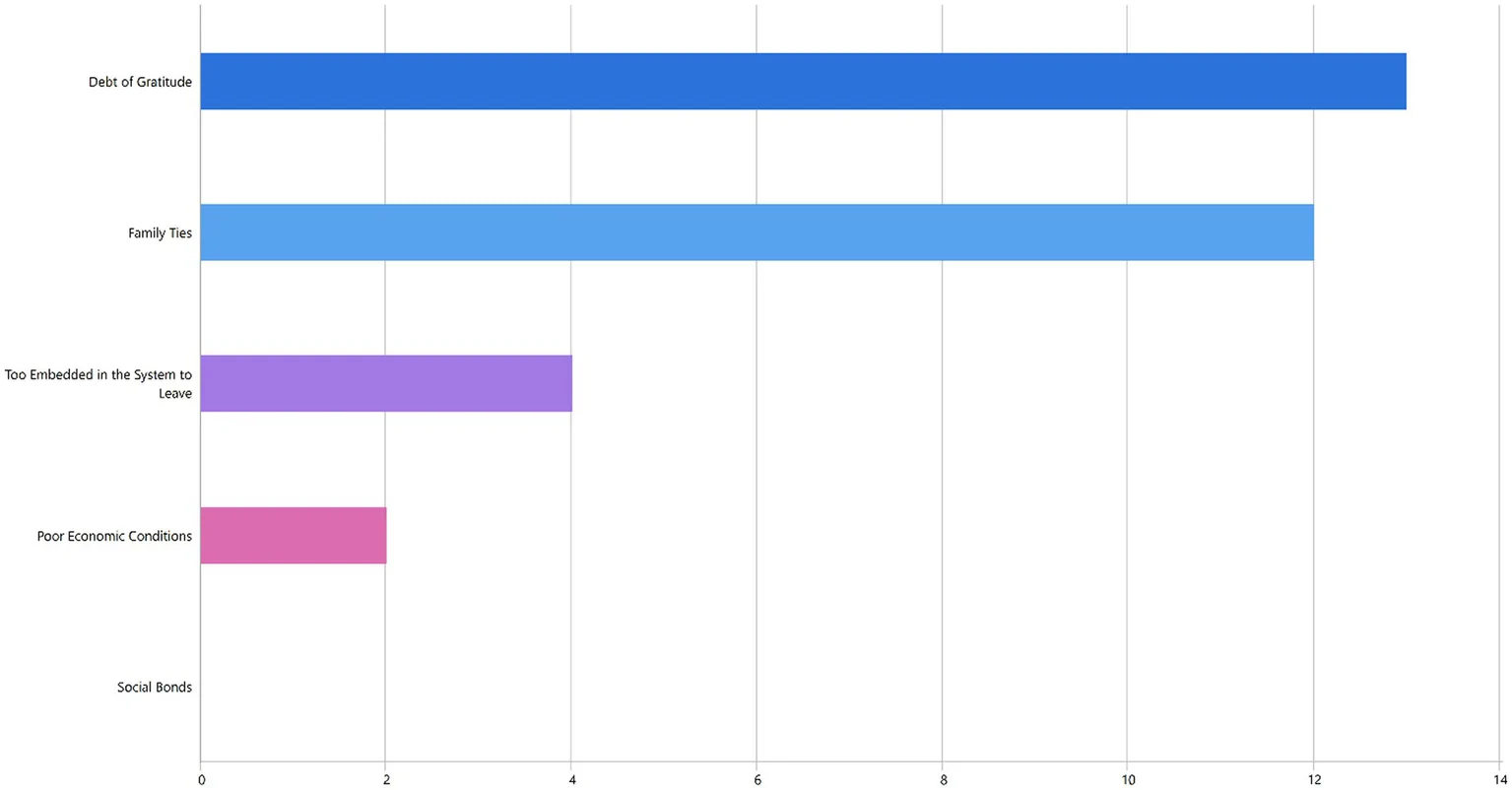
Category of Family, Social Ties and Economic Hardship. This figure ranks ‘‘Debt of Gratitude’’ and ‘‘Family Ties’’ as the leading socio-cultural motivators for engineers remaining within the domestic organizational system.

“I did three and a half years compulsory service, then stayed; it’s both habit and loyalty.” — M10.

“My mandatory service period is ending, but I’m not considering leaving. My family is here, I have children; I’ve put down roots now.” — M11.

“We work intensely, I do not have a clear plan for the future, but I’m here now, and I feel like I’ll continue here.” — M3.

As is evident from Figure 8, “Systemic Support Adjustment and Need for a Detailed Orientation and Supervisorship Programme” codes stand out as two important codes in this category. Participants emphasise the need for fundamental and detailed improvements to the current orientation system, as well as systemic regulations in areas such as guidance, counselling, and access to technical and psychological support resources. In parallel with this situation, they also mention that it would be beneficial to organise an internship programme. They believe that this internship programme would serve as a good orientation for both pre- and post-internship individuals. Another important issue is that the work culture acquired by participants through education and internship opportunities abroad and the projects they have participated in does not work in state-owned institutions, and this situation causes many problems. In this context, the two codes that stand out are “Structural, Bureaucratic and Hierarchical Slowness” and “Professionalism Requirements/Demands.” Similarly, the codes “Market and Organisational Culture Incompatibility,” “Orientation Support for Newcomers,” and “Privatisation Demands” are also highlighted as important issues by the cases. Some sample statements from cases:

**Figure 8:**
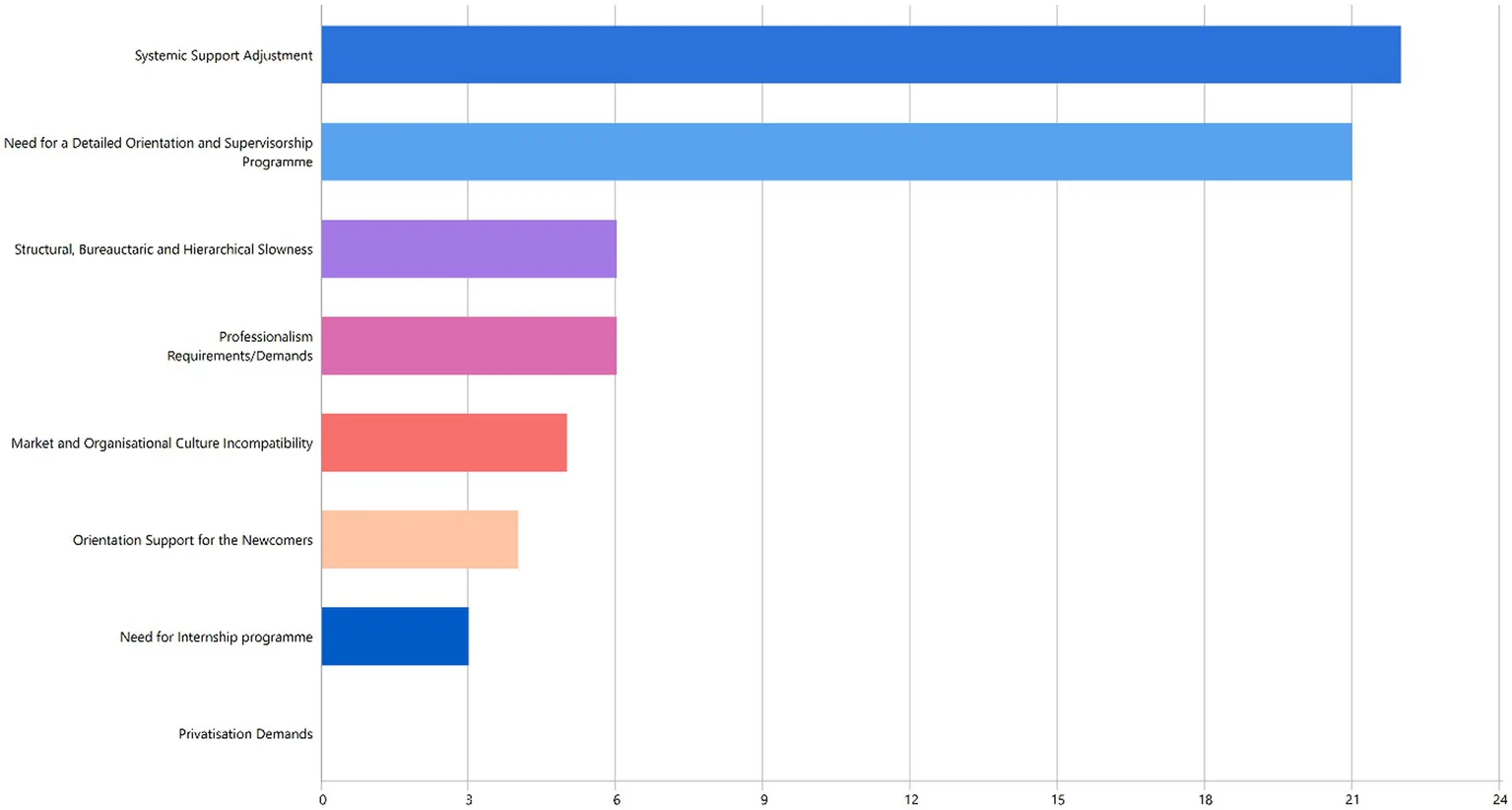
Category of Institutional, Political and Economic Factors Connected Bureaucratic Structure (Systemic Support). This visualization highlights the critical need for ‘‘Systemic Support Adjustment’’ and ‘‘Detailed Orientation and Supervisorship Programmes’’ to facilitate organizational integration.

“Professionalism abroad is very high; academia and companies coordinate incredibly well. In Turkey, however, cooperation between academia and CC is weak, limiting our work’s impact.” — M10.

“Structural and hierarchical slowness within the institution means processes are so long that newcomers lose energy.” — M7.

‘If the company had links with academia, we would write our theses on topics relevant to the institution and adapt more quickly when we returned.’ — M8.

In this visual MAXQDA map, Figure 9 demonstrates four main relationships: ‘System Awareness and Adaptation to the Academic System - Conscious Choice, Need for a Detailed Orientation and Supervisorship Programme - Systemic Support Adjustment, Family Ties - Compensation Model ‘Implicit Obligation’, Vocational Training - Competence Development - Specialisation and Incompatible Field Experience’. In this map, the direction of the arrows reveals the relationships between the codes and their directions, while the thick and dashed lines reveal the intensity of the relationship. In this context, System Awareness and Academic Development Barrier appear as a distinct pattern among the participants. Most participants have a system awareness about academy but due to their scholarship they could not be one part of the academy and gave up their further academic development such as PhD. Again, the connections between the codes in this framework show that this situation is directly related to the code ‘academic choice orientation’. They are aware of the fact that the scholarship they accept both in scholarship applications, in post-acceptance university choices and in the following times will cause the participants to act in line with their main choices after a while. Another important pattern that stands out in the map and is seen in all cases as a literal replication is the relationship between emotional attachment, obligation, and decision-making pressure. In this framework, ‘Debt of Gratitude - Compensation Model ‘Implicit Obligation’ - Family Ties’ shows a strong relationship. In the map, the “Systemic Support Adjustment” code appears as the area with the most intense relations. In terms of academic, organisational and market adaptation, the participants state that the existing support mechanism should be updated and a more inclusive mechanism that addresses all these dimensions at the same time and together should be established. Again, as a reflection of this lack of systemic support, all cases intensively reflected the code “Specialisation and Incompatible Field Experience” on the map. This situation has led to a compulsory development in terms of both skill development and vocational training within the framework of the conditions in which they are in privileged cases, and they have gained new skills and experiences in fields other than their own specialisation. This situation they have experienced has found a place on the map with the code ‘Reality Shock’ and has become an acceptance. As a result of this acceptance, almost all of the cases continue to work in their companies despite the end of their compulsory service period, and the cases explain this situation with the code ‘Too Embedded in the System to Leave’.

**Figure 9:**
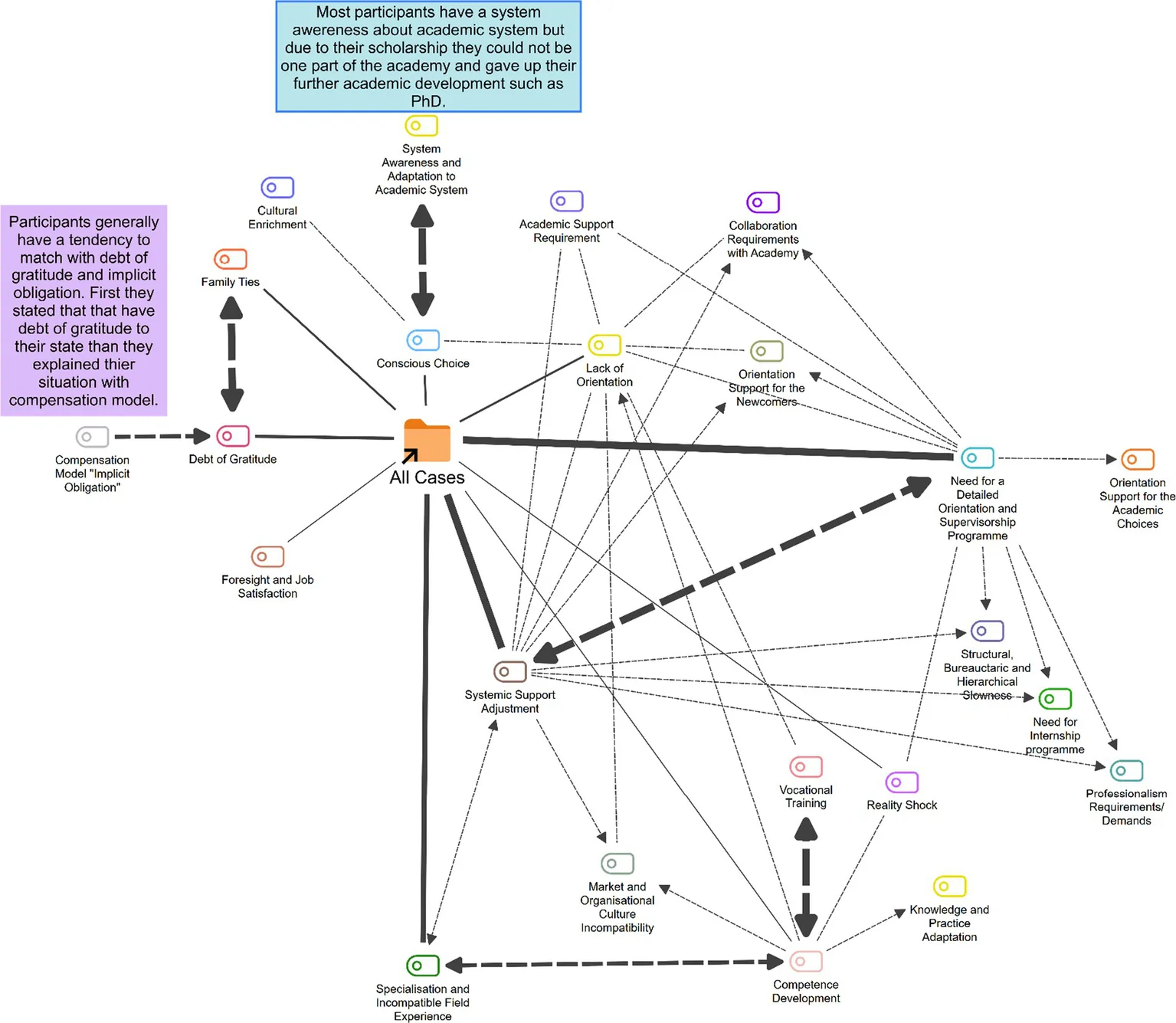
Visual map of all cases. This MAXQDA-generated map illustrates the complex relationships among the most strongly inter-related codes, such as the link between ‘‘Debt of Gratitude’’ and the ‘‘Compensation Model,’’ and the impact of ‘‘Reality Shock’’ on adaptation.

## 6 Discussion

The present study examines the integration and adaptation dynamics experienced by CC engineers who returned to Turkey under the scholarship bond. This is achieved through case study and qualitative content analysis. The findings reflect the contradictions between these engineers’ world-class academic and professional experience and CC’s local working environment. The data obtained present a discussion filtered through the theoretical frameworks of organisational socialisation, reverse brain drain, knowledge transfer, and institutional logics.

In the study, experiences emerging under codes such as ‘lack of orientation’ and ‘reality shock’ make significant contributions to Van Maanen and Schein's (1979) organisational socialisation theory. This theory posits that organisational socialisation processes are of paramount importance for the adoption of organisational norms and roles by new employees. As posited by AlZamil and Alyafi (2022), the process of organisational socialisation has been demonstrated to have a positive impact on organisational commitment. However, the present study demonstrates that the absence of a structured organisational socialisation process resulted in participants relying on their individual ‘meaning-making’ processes (Louis, 1980; Hoffmann and Thyregod Rasmussen, 2022). This standpoint is at odds with the existing literature, which emphasises the significance of organisational socialisation for knowledge transfer (Khalil et al., 2021; Moeller, 2024; Srinivas and Vanka, 2022; de Andrade and Benfica, 2023). The absence of formal institutional socialisation procedures engenders considerable challenges in the adaptation of returning engineers, thereby impeding the organisation’s capacity for knowledge transfer. Informal social learning and peer support mechanisms within the organisation act as a buffer for institutional socialisation. CC relies on its informal social capital (experienced human resources) within the context of institutional socialisation. Nevertheless, this reliance on informal mechanisms gives rise to disparate integration experiences among engineers returning to their country of origin and the inefficient utilisation of skilled human resources.

Building on these insights, the research extends the existing body of knowledge on reverse brain drain approaches by exploring factors that extend beyond economic motivations. The research reveals that engineers who consciously choose to return are influenced by socio-cultural and ethical factors such as “debt of gratitude,” “family ties” and “compensation model (implicit obligation),” which shape their decisions to return and remain. The bond thus operates on two registers at once: as a formal service-or-repay contract, and as a moral debt that participants describe in the language of gratitude. This doubling helps explain why obligation proved such a durable retention force. This situation suggests that the institution is capable of attracting talent through scholarship, which is consistent with its macro-level policy. However, it faces challenges in effectively integrating this talent at the micro-level. The institution faces the challenge of creating a conducive working environment that can effectively leverage the unique expertise acquired by returning engineers through their international experiences. The majority of participants frequently cited a discrepancy between their areas of expertise and the work they undertook upon their return. This situation is particularly concerning in terms of its impact on the participants’ professional satisfaction. Moreover, while the scholarship model is indeed effective in facilitating return, it concomitantly engenders an “academic development barrier.” The findings, which include specialisation, mismatched field experience and professional training, also highlight missed opportunities for innovation, productivity and strategic advantage that the reverse brain drain policy aims to achieve. The failure to leverage world-class knowledge in accordance with organisational requirements may also compromise national objectives related to innovation and energy independence.

Contrary to the perspective that defines brain drain as a loss of talent and expertise for the source country, the research demonstrates the existence of a complex “brain gain” process that encourages return. Furthermore, this process evokes the phenomena of brain circulation (Saxenian, 2002b, 2002c), through which engineers transfer technology and knowledge via networks they establish with their home countries, and ‘knowledge networks’ (Giannetti et al., 2015) and ‘diaspora networks’ (Tung and Lazarova, 2006) that support economic development. However, the findings of this study also reflect the counterpoint that these strong ties and obligations may lead engineers to remain due to obligation rather than professional adaptation.

The finding of ‘specialisation and mismatched field experience’ contrasts with the fundamental assumptions of knowledge transfer and innovation theory (Nonaka, 1994), which examines the transfer of knowledge acquired abroad to local processes. Consequently, the inability to directly and optimally apply specific knowledge and skills acquired abroad demonstrates that knowledge transfer cannot be achieved and does not support innovation. In a similar vein, Singh et al. (2023) underscore the notion that knowledge transfer from multinational corporations serves to bolster innovation. However, it has been concluded that CC exhibits a low “absorptive capacity” (Cohen and Levinthal, 1990) regarding the specialised knowledge contributed by engineers. A disparity between the globally acquired expertise and CC’s absorptive capacity and operational needs can impede the efficacy of knowledge transfer. Moreover, the finding of ‘system awareness and adaptation to the academic system’ corroborates the observation that engineers returning due to obligations encounter difficulties in continuing their academic work from another perspective. In this particular instance, the absence of an ongoing relationship between the universities where the participants pursued their studies and the companies where they acquired professional experience, combined with the limitation of collaboration with foreign universities and scientists, exacerbates the challenges encountered. Jonkers and Tijssen (2008) show that returnees who sustain ties with their host-country institutions maintain higher levels of research collaboration and scientific productivity, a mechanism largely absent in the present case. This phenomenon exemplifies the potential consequences of the reverse brain drain strategy, which endeavours to facilitate the international mobility of skilled professionals to acquire international experience. However, the absence of a conducive environment for utilising this knowledge and experience can lead to a situation where the intended benefits are not realised. Instead, what was intended as brain gain may amount to a form of ‘brain loss’.

In this context, the institutional logics approach, which forms the basis for addressing the fundamental conflict between the ‘public/bureaucratic logic’ prevalent in the organisation due to CC being a public economic enterprise and the ‘global education/market logic’ represented by the returning engineers (Thornton and Ocasio, 2008), strongly aligns with the findings. Participants frequently highlighted issues such as ‘structural, bureaucratic and hierarchical slowness,’ ‘professionalism requirements/demands,’ and ‘market and organisational culture incompatibility.’ This phenomenon is indicative of a profound discord between the global work culture and project experience acquired abroad and the operational realities of a state-owned institution. The reference to ‘privatisation demands’ reflects the participants’ growing awareness of this fundamental logical conflict. Engineers who have internalised a ‘global rationality’ (Meyer and Rowan, 1977) that prioritises efficiency, innovation, and merit state that their professional autonomy and ability to apply best practices are severely constrained by an entrenched ‘bureaucratic logic’. Moreover, the findings indicate a decoupling (Meyer and Rowan, 1977) between policy and practice, whereby formal procedures deviate from prevailing organisational practices. This discord has engendered a sense of disillusionment among returnees and has impeded CC from deriving full benefit from its scholarship investments. This decoupling, therefore, provides an accurate example of the institution’s functioning under various and potentially conflicting logics. The research presents a substantial empirical case for understanding the “decoupling” and internal conflicts that arise from the interaction between “global education/market logics” and “public/bureaucratic logics” in a hybrid organisational context, such as CC.

This research addresses the gap identified in the introduction by examining reverse brain drain and post-return adaptation processes at the micro level (through institutional analysis) using a qualitative case study of engineers who returned to CC under the scholarship bond, employing an institutional logics approach. The presence of engineers who have acquired world-class knowledge and returned with a more market-oriented mindset can result in the emergence of logical contradictions within an organisation that predominantly operates within a public sector framework. These contradictions arise from a hybrid organisational structure in the sense that public and private logics are intertwined. The process of translating globally standardised skills acquired through education into local expertise requires adaptation, knowledge transfer, and the establishment/development of diaspora networks.

## 7 Conclusion

This study set out to examine how the socio-psychological experiences of scholarship-funded engineers evolve after their return, as they adapt to their home organisation and its competing institutional logics. The findings reveal a consistent trajectory: engineers move from an initial period of disorientation and reality shock—shaped by inadequate orientation and expertise-role mismatches—toward a form of constrained accommodation, in which socio-cultural ties and ethical obligations sustain organisational commitment even where professional satisfaction remains limited.

These findings carry implications across the four theoretical frameworks employed in this study. Within organisational socialisation theory, the absence of structured resocialisation procedures emerges not merely as a practical shortcoming but as an institutional failure to recognise that returning engineers are not newcomers in the conventional sense—they are professionals whose prior knowledge complicates rather than facilitates re-entry. From a reverse brain drain perspective, the findings demonstrate that the return decision and the retention decision rest on fundamentally different motivational foundations: while scholarship obligations and socio-cultural attachments drive return, professional satisfaction and role alignment determine whether that return translates into genuine organisational contribution. The knowledge transfer analysis reveals a persistent gap between CC’s absorptive capacity and the specialised expertise its scholarship programme was designed to cultivate. Finally, the institutional logics lens captures the structural source of these tensions—a collision between the global market logic engineers internalised abroad and the public-bureaucratic logic that governs CC’s operations, which current institutional arrangements do not adequately mediate.

Taken together, the findings suggest that scholarship-bound return programmes cannot rely on obligation alone to convert brain return into brain gain. What this study adds to that general point is a specification of where absorption breaks down. Three mechanisms stand out in the data. The absence of structured resocialisation shifts the burden of integration onto individual sense-making, with outcomes the institution neither tracks nor corrects. Expertise-role mismatch operates systematically rather than incidentally, rooted in placement practices that answer staffing needs before specialisation. Moreover, the co-existence of bureaucratic and market logics remains unmediated: returnees have no legitimate channel through which their market-oriented practices can enter organisational routines. Each recommendation in the following section answers one of these documented failure points, which is what grounds their applicability to CC and to structurally similar scholarship-bound programmes elsewhere.

### 7.1 Recommendations

Drawing on the study’s findings, the following recommendations are directed at institutional managers and policymakers responsible for the design and oversight of scholarship-based repatriation programmes. Each recommendation targets a specific gap identified in the adaptation experiences of returning engineers at CC.

- Scholarship frameworks that acknowledge debt of gratitude dynamics and family attachment factors will better reflect the actual motivations that sustain engineers’ long-term organisational commitment.
- CC should introduce a structured, phased socialisation programme for returning engineers, covering both organisational norms and role expectations. A well-designed orientation process—one that extends beyond a single introductory meeting—would substantially reduce the reality shock that currently characterises the early post-return period.
- Talent placement decisions should actively align engineers’ areas of specialisation with their assigned responsibilities. The current pattern of expertise-role mismatch not only undermines professional satisfaction but represents an institutional failure to leverage the knowledge CC’s scholarship programme was designed to develop.
- Reducing bureaucratic rigidity and creating space for market-oriented working practices would increase CC’s absorptive capacity—its ability to integrate and apply the globally acquired expertise of returning engineers. This is a prerequisite for translating scholarship investments into organisational and sectoral innovation.
- Enhanced academic and professional networks necessitate developing mechanisms enabling engineers’ continued academic development and encouraging foreign university cooperation.

Implemented together, these measures would improve both the professional experience of returning engineers and CC’s capacity to fulfil the strategic objectives for which its scholarship programme was established.

### 7.2 Limitations and future work

This study carries five substantive limitations that should be considered when interpreting the findings. First, the single-case design—focused exclusively on engineers at CC—restricts the generalisability of the findings beyond this institutional context and the oil and gas sector. For this reason, we frame the study’s contribution in terms of analytical transferability rather than empirical generalisability: the mechanisms documented here (unmanaged resocialisation, systematic expertise-role mismatch, unmediated logic conflict) offer a conceptual vocabulary that can be carried to structurally similar scholarship-bound programmes and examined there, rather than assumed to hold. Second, all participants were male, reflecting the gender composition of CC’s scholarship cohort during the study period; conclusions about gender dynamics in reverse brain drain adaptation, therefore, remain beyond the scope of this study. Third, the retrospective nature of the interview data introduces the possibility of recall bias, as participants were asked to reflect on experiences that, in some cases, occurred several years earlier. Fourth, the study rests on the accounts of the repatriated engineers themselves. The perspectives of managers, mentors, and human resources staff were not independently examined, which limits the extent to which claims about institutional practice can be triangulated. When participants describe organisational procedures, those descriptions reflect their experience of the system rather than how the system is presented by its administrators. Fifth, the sample comprises engineers who took the service arm of the scholarship bond and were still employed at CC at the time of the interviews. Scholars who repaid the bond and remained abroad, and returnees who left the institution once their service ended, fall outside the study; their experiences would show the same arrangement from its other exits. Future research would benefit from comparative designs spanning multiple organisations and sectors, as well as longitudinal approaches that track adaptation trajectories from the point of return rather than retrospectively.

## Data Availability

The raw data supporting the conclusions of this article will be made available by the authors, without undue reservation.

## References

[ref1] AlZamil B. S. Alyafi R. S. (2022). Organizational socialization applicability, measurements, and theories: a systematic review and guideline for future research. Int. J. Bus. Manag. Econ. Rev. 5, 219–239. doi: 10.35409/ijbmer.2022.3424

[ref2] Argote L. Ingram P. (2000). Knowledge transfer: a basis for competitive advantage in firms. Organ. Behav. Hum. Decis. Process. 82, 150–169. doi: 10.1006/obhd.2000.2893

[ref3] Artuç E. Docquier F. Özden Ç. Parsons C. (2015). A global assessment of human capital mobility: the role of non-OECD destinations. World Dev. 65, 6–26. doi: 10.1016/j.worlddev.2014.04.004

[ref4] Astor A. Akhtar T. Matallana M. A. Muthuswamy V. Olowu F. A. Tallo V. . (2005). Physician migration: views from professionals in Colombia, Nigeria, India, Pakistan, and the Philippines. Soc. Sci. Med. 61, 2492–2500. doi: 10.1016/j.socscimed.2005.05.003, 15953667

[ref5] Bartolini L. Gropas R. Triandafyllidou A. (2017). Drivers of highly skilled mobility from southern Europe: escaping the crisis and emancipating oneself. J. Ethn. Migr. Stud. 43, 652–673. doi: 10.1080/1369183x.2016.1249048

[ref6] Bauer T. N. Erdogan B. (2011). “Organizational socialization: the effective onboarding of new employees,” in APA Handbook of Industrial and Organizational Psychology, Vol. 3. Maintaining, Expanding, and Contracting the Organization, ed. Zedeck S. (Washington, DC: American Psychological Association), 51–64. doi: 10.1037/12171-002

[ref7] Beine M. Docquier F. Oden-Defoort C. (2011). A panel data analysis of the brain gain. World Dev. 39, 523–532. doi: 10.1016/j.worlddev.2010.03.009

[ref8] Bokayev B. Torebekova Z. Davletbayeva Z. (2020). Preventing brain drain: Kazakhstan’s presidential “bolashak” scholarship and government regulations of intellectual migration. Public Policy Adm. 19, 25–35. doi: 10.5755/j01.ppaa.19.3.27764

[ref9] Bollard A. McKenzie D. Morten M. Rapoport H. (2011). Remittances and the brain drain revisited: the microdata show that more educated migrants remit more. World Bank Econ. Rev. 25, 132–156. doi: 10.1093/wber/lhr013

[ref10] Bosetti V. Cattaneo C. Verdolini E. (2015). Migration of skilled workers and innovation: a European perspective. J. Int. Econ. 96, 311–322. doi: 10.1016/j.jinteco.2015.04.002

[ref11] Cebolla-Boado H. Hu Y. Soysal Y. N. (2017). Why study abroad? Sorting of Chinese students across British universities. Br. J. Sociol. Educ. 39, 365–380. doi: 10.1080/01425692.2017.1349649, 37339054

[ref12] Chacko E. (2007). From brain drain to brain gain: reverse migration to Bangalore and Hyderabad, India’s globalizing high tech cities. GeoJournal 68, 131–140. doi: 10.1007/s10708-007-9078-8

[ref13] Chao G. T. O’Leary-Kelly A. M. Wolf S. Klein H. J. Gardner P. D. (1994). Organizational socialization: its content and consequences. J. Appl. Psychol. 79, 730–743. doi: 10.1037/0021-9010.79.5.730

[ref14] Cohen W. M. Levinthal D. A. (1990). Absorptive capacity: a new perspective on learning and innovation. Adm. Sci. Q. 35, 128–152. doi: 10.2307/2393553

[ref15] de Andrade R. D. Benfica V. C. (2023). The evolution of knowledge sharing and innovation: exploring epistemological reflections. Contribuciones A Las Ciencias Sociales 16, 8034–8052. doi: 10.55905/revconv.16n.7-228

[ref16] Desai M. A. Kapur D. McHale J. Rogers K. (2009). The fiscal impact of high-skilled emigration: flows of Indians to the US. J. Dev. Econ. 88, 32–44. doi: 10.1016/j.jdeveco.2008.01.008

[ref17] Di Maria C. Lazarova E. A. (2012). Migration, human capital formation, and growth: an empirical investigation. World Dev. 40, 938–955. doi: 10.1016/j.worlddev.2011.11.011

[ref18] Docquier F. Rapoport H. (2012). Globalization, brain drain, and development. J. Econ. Lit. 50, 681–730. doi: 10.1257/jel.50.3.681

[ref19] Dulam T. Franses P. H. (2015). Emigration, wage differentials, and brain drain: the case of Suriname. Appl. Econ. 47, 2339–2347. doi: 10.1080/00036846.2015.1005826

[ref20] Dustmann C. Fadlon I. Weiss Y. (2011). Return migration, human capital accumulation, and the brain drain. J. Dev. Econ. 95, 58–67. doi: 10.1016/j.jdeveco.2010.04.006

[ref21] Eggert W. Krieger T. Meier V. (2010). Education, unemployment, and migration. J. Public Econ. 94, 354–362. doi: 10.1016/j.jpubeco.2010.01.005, 38826717

[ref22] Facchini G. Mayda A. M. (2009). Does the welfare state affect individual attitudes toward immigrants? Evidence across countries. Rev. Econ. Stat. 91, 295–314. doi: 10.1162/rest.91.2.295

[ref24] Friedland R. Alford R. R. (1991). “Bringing society back in: symbols, practices, and institutional contradictions,” in The New Institutionalism in Organizational Analysis, eds. Powell W. W. DiMaggio P. J. (Chicago: University of Chicago Press), 232–263.

[ref25] Giannetti M. Liao G. Yu X. (2015). The brain gain of corporate boards: evidence from China. J. Finance 70, 1629–1682. doi: 10.1111/jofi.12198

[ref26] Gibson J. McKenzie D. (2011). The microeconomic determinants of emigration and return migration of the best and brightest: evidence from the Pacific. J. Dev. Econ. 95, 18–29. doi: 10.1016/j.jdeveco.2009.11.002

[ref27] Güngör N. D. Tansel A. (2007). Brain drain from Turkey: The case of Professionals Abroad (IZA Discussion Paper No. 2617). Bonn, Germany: IZA Institute of Labor Economics.

[ref28] Güngör N. D. Tansel A. (2014). Brain drain from Turkey: return intentions of skilled migrants. Int. Migr. 52, 208–226. doi: 10.1111/imig.12013

[ref29] Ha W. Yi J. Zhang J. (2016). Brain drain, brain gain, and economic growth in China. China Econ. Rev. 38, 322–337. doi: 10.1016/j.chieco.2015.02.005

[ref30] Hoffmann J. Thyregod Rasmussen M. (2022). Expanding the theoretical landscape of organizational socialization research: institutionalism and beyond. Commun. Lang. Work. 8, 4–16. doi: 10.7146/claw.v8i2.135109

[ref31] Hussain S. M. (2015). Reversing the brain drain: is it beneficial? World Dev. 67, 310–322. doi: 10.1016/j.worlddev.2014.10.023

[ref32] Ibrahim H. Al Sharif F. Z. Satish K. P. Hassen L. Nair S. C. (2019). Should I stay or should I go now? The impact of “pull” factors on physician decisions to remain in a destination country. Int. J. Health Plann. Manag. 34, e1909–e1920. doi: 10.1002/hpm.2819, 31169326

[ref33] Ifanti A. A. Argyriou A. A. Kalofonou F. H. Kalofonos H. P. (2014). Physicians’ brain drain in Greece: a perspective on the reasons why and how to address it. Health Policy 117, 210–215. doi: 10.1016/j.healthpol.2014.03.014, 24794987

[ref34] Jonkers K. Cruz-Castro L. (2013). Research upon return: the effect of international mobility on scientific ties, production and impact. Res. Policy 42, 1366–1377. doi: 10.1016/j.respol.2013.05.005

[ref35] Jonkers K. Tijssen R. (2008). Chinese researchers returning home: impacts of international mobility on research collaboration and scientific productivity. Scientometrics 77, 309–333. doi: 10.1007/s11192-007-1971-x

[ref36] Khalil K. Asgher U. Khalil M. Khawaja K. Ayaz Y. Nazir S. (2021). “An empirical study on organizational socialization and its relationship with employees’ age and the knowledge management,” in Advances in Neuroergonomics and Cognitive Engineering. AHFE 2020. Advances in Intelligent Systems and Computing, eds. Ayaz H. Asgher U., vol. 1201 (Cham: Springer).

[ref37] Klafki W. (2001). “Hermeneutische Verfahren in der Erziehungswissenschaft,” in Einführung in die pädagogische Hermeneutik. Mit einem Beitrag von Wolfgang Klafki, eds. Rittelmeyer C. Parmentier M. (Darmstadt: Wissenschaftliche Buchgesellschaft), 125–148.

[ref39] Köser-Akçapar S. (2006). Do brains really going down the drain? Rev. Eur. Migr. Int. 22, 79–107. doi: 10.4000/remi.3281

[ref40] Koska O. A. Saygın P. O. Çağatay S. Artal-Tur A. (2013). International migration, remittances, and the human capital formation of Egyptian children. Int. Rev. Econ. Financ. 28, 38–50. doi: 10.1016/j.iref.2013.05.006

[ref41] Kwok V. Leland H. (1982). An economic model of the brain drain. Am. Econ. Rev. 72, 91–100.

[ref42] Li X. McHale J. Zhou X. (2017). Does brain drain lead to institutional gain? World Econ. 40, 1454–1472. doi: 10.1111/twec.12407

[ref43] Lincoln Y. S. Guba E. G. (1985). Naturalistic Inquiry. Newbury Park, CA: Sage.

[ref44] Louis M. R. (1980). Surprise and sense making: what newcomers experience in entering unfamiliar organizational settings. Adm. Sci. Q. 25, 226–251. doi: 10.2307/2392453, 10247029

[ref45] Lowell B. Findlay A. (2002). Migration of Highly Skilled Persons from Developing Countries: Impact and Policy Responses: Synthesis Report. Geneva, Switzerland: International Labour Office.

[ref46] Meyer J. W. Rowan B. (1977). Institutionalized organizations: formal structure as myth and ceremony. Am. J. Sociol. 83, 340–363. doi: 10.1086/226550

[ref47] Michaelis A. R. (1990). Brain drain and brain gain. Interdiscip. Sci. Rev. 15, 193–195. doi: 10.1179/isr.1990.15.3.193

[ref48] Moeller M. (2024). “(Re)socialisation theory,” in A Guide to Key Theories for Human Resource Management Research. (eds). Hutchings K. Michailova S. Wilkinson A. (Cheltenham, United Kingdom: Edward Elgar Publishing), 224–230. doi: 10.4337/9781035308767.ch28

[ref49] Mountford A. (1997). Can a brain drain be good for growth in the source economy? J. Dev. Econ. 53, 287–303. doi: 10.1016/S0304-3878(97)00021-7, 12348295

[ref50] Nguyen T. L. Yeoh B. S. A. Toyota M. (2006). Migration and the well-being of the ‘left behind in Asia’. Asian Popul. Stud. 2, 37–44. doi: 10.1080/17441730600700507

[ref51] Nonaka I. (1994). A dynamic theory of organizational knowledge creation. Organ. Sci. 5, 14–37. doi: 10.1287/orsc.5.1.14, 19642375

[ref52] Okeke E. N. (2013). Brain drain: do economic conditions “push” doctors out of developing countries? Soc. Sci. Med. 98, 169–178. doi: 10.1016/j.socscimed.2013.09.010, 24331896

[ref53] Page J. Plaza S. (2006). Migration remittances and development: a survey of the global evidence. J. Afr. Econ. 15, 245–336. doi: 10.1093/jae/ejl035

[ref54] Patton M. Q. (2002). Qualitative Research & Evaluation Methods. Thousand Oaks, CA: Sage.

[ref55] Poppe A. Jirovsky E. Blacklock C. Laxmikanth P. Moosa S. Maeseneer J. D. . (2014). Why sub-Saharan African health workers migrate to European countries that do not actively recruit: a qualitative study post-migration. Glob. Health Action 7:Article 24071. doi: 10.3402/gha.v7.24071, 24836444 PMC4021817

[ref56] Punch K. F. (2005). Introduction to Social Research: Quantitative and Qualitative Approaches. London, United Kingdom: Sage.

[ref57] Purnomo S. Utami I. W. Gill A. A. (2022). The impact of organizational socialization and career development on intention to change work in public companies in Pakistan. J. Manaj. Bisnis 13, 108–120. doi: 10.18196/mb.v13i1.14102

[ref58] Reichers A. E. (1987). An interactionist perspective on newcomer socialization rates. Acad. Manag. Rev. 12, 278–287. doi: 10.2307/258535

[ref59] Sahay P. (2014). Design thinking in talent acquisition: a practitioner’s perspective. Strateg. HR Rev. 13, 170–180. doi: 10.1108/shr-04-2014-0027

[ref60] Saldaña J. (2021). The Coding Manual for Qualitative Researchers. Thousand Oaks, CA: Sage Publications.

[ref61] Saxenian A. (2002a). Brain circulation: how high-skill immigration makes everyone better off. Brook. Rev. 20, 28–31. doi: 10.2307/20081018

[ref62] Saxenian A. (2002b). Silicon Valley’s new immigrant high-growth entrepreneurs. Econ. Dev. Q. 16, 20–31. doi: 10.1177/0891242402016001003

[ref63] Saxenian A. (2002c). Transnational communities and the evolution of global production networks: the cases of Taiwan, China and India. Ind. Innov. 9, 183–202. doi: 10.1080/1366271022000034453

[ref64] Saxenian A. (2005). From brain drain to brain circulation: transnational communities and regional upgrading in India and China. Stud. Comp. Int. Dev. 40, 35–61. doi: 10.1007/bf02686293

[ref65] Singh S. Singh S. Dhir S. (2023). The evolving relationship of entrepreneurship, technology, and innovation: a topic modeling perspective. Int. J. Entrep. Innov. 27, 91–110. doi: 10.1177/14657503231179597

[ref66] Sjaastad L. A. (1962). The costs and returns of human migration. J. Polit. Econ. 70, 80–93. doi: 10.1086/258726, 11892910

[ref67] Srinivas J. Vanka S. (2022). “Is organizational socialization moving towards digital socialization? – a perspective,” in Academy of Management Proceedings.

[ref68] Taylor E. J. (1999). The new economics of labour migration and the role of remittances in the migration process. Int. Migr. 37, 63–88. doi: 10.1111/1468-2435.00066, 12322077

[ref69] Tharenou P. Seet P. S. (2014). China’s reverse brain drain: regaining and retaining talent. Int. Stud. Manag. Organ. 44, 55–74. doi: 10.2753/IMO0020-8825440203

[ref70] Thornton P. H. Ocasio W. (2008). “Institutional logics,” in The Sage Handbook of Organizational Institutionalism, (eds). Greenwood R. Oliver C. Suddaby R. Sahlin-Andersson K. (London: Sage), 99–128. doi: 10.4135/9781849200387.n4, 42549636

[ref71] Traeger C. Haski-Leventhal D. Alfes K. (2021). Extending organizational socialization theory: empirical evidence from volunteer work for refugees in France and Australia. Hum. Relat. 75, 1140–1166. doi: 10.1177/00187267211006451

[ref72] Tung R. L. Lazarova M. (2006). Brain drain versus brain gain: an exploratory study of ex-host country nationals in central and East Europe. Int. J. Hum. Resour. Manag. 17, 1853–1872. doi: 10.1080/09585190600999992

[ref73] Van Maanen J. Schein E. H. (1979). Toward a theory of organizational socialization. Res. Organ. Behav. 1, 209–264.

[ref74] Vizi E. S. (1993). Reversing the brain drain from eastern European countries: the “push” and “pull” factors. Technol. Soc. 15, 101–109. doi: 10.1016/0160-791x(93)90029-n

[ref75] Williams A. M. (2007). International labour migration and tacit knowledge transactions: a multi-level perspective. Glob. Netw. 7, 29–50. doi: 10.1111/j.1471-0374.2006.00155.x

[ref76] Yılmaz-Şener M. Türgen G. (2018). Kesin Dönüş Mü, Yoksa Tekrar Göç Etme Hazırlığı Mı? Nitelikli Türk Göçmenlerin Almanya ve Amerika’dan Geriye Göçü. Sosyoloji Araştırmaları Dergisi 21, 311–377. doi: 10.18490/sosars.419072

[ref77] Yin R. K. (2003). Case Study Research: Design and Methods. Applied Social Research Methods, vol. 5. 3rd Edn London: Sage Publications.

[ref78] Zweig D. (2006). Competing for talent: China’s strategies to reverse the brain drain. Int. Labour Rev. 145, 65–90. doi: 10.1111/j.1564-913x.2006.tb00010.x

[ref79] Zweig D. Tsai K. S. Singh A. D. (2021). Reverse entrepreneurial migration in China and India: the role of the state. World Dev. 138:105192. doi: 10.1016/j.worlddev.2020.105192, 38826717

