# Correction: Repatriation, identity, and organisational adaptation: a case study on reverse brain drain and socio-psychological drivers in Turkey's energy sector

**DOI:** 10.3389/fpsyg.2026.2023199

**Published:** 2026-10-27

**Authors:** Cihan Durmuşkaya, Mustafa Abdül Metin Dinçer, Onur Metin, Akin Özdemir

**Affiliations:** 1 Department of Labour Economics and Industrial Relations, Faculty of Political Sciences, Sakarya University, Sakarya, Türkiye; 2 Human Resources Management Program, Department of Management and Organisation, Geyve Vocational School, Sakarya University of Applied Sciences, Sakarya, Türkiye; 3 Sakarya Economic and Social Research Center (SESAM), Sakarya University, Sakarya, Türkiye

**Keywords:** institutional logics, knowledge transfer, organisational socialisation, qualitative case study, reality shock, return migration, scholarship-bound mobility

In the published article, there was a mistake in Supplementary Table 1. To ensure strict participant confidentiality and prevent any deductive disclosure risks within the studied organisation, the specific ages, exact years of experience, and specific branch locations of the participants have been anonymised into broader ranges. The corrected Supplementary Table 1 appears below.

**Supplementary Table 1:** Participants profiles.

Case	Age	Education	Job title	Experience in CC	Location in the CC	Duration of Interviews
M1	30-35	MS	Junior Engineer	1-5 years	CC	40-50
M2	35-40	Phd	Senior Engineer	10-15 years	CC	70-80
M3	35-40	MS	Mid-level Engineer	1-5 years	CC	50-60
M4	35-40	MS	Senior Engineer	5-10 years	CC	50-60
M5	30-35	MS	Senior Engineer	5-10 years	CC	50-60
M6	35-40	Phd	Senior Engineer	10-15 years	CC	60-70
M7	35-40	MS	Senior Engineer	10-15 years	CC	70-80
M8	35-40	MS	Senior Engineer	10-15 years	CC	60-70
M9	35-40	MS	Senior Engineer	10-15 years	CC	60-70
M10	35-40	MS	Senior Engineer	5-10 years	CC	60-70
M11	35-40	MS	Senior Engineer	5-10 years	CC	70-80
M12	35-40	MS	Mid-level Engineer	5-10 years	CC	40-50

The original version of this article has been updated.

