# Supplementary material for: Repatriation, identity, and organisational adaptation: a case study on reverse brain drain and socio-psychological drivers in Turkey’s energy sector
Source: Front Psychol. 2026 Oct 2;17:1802059. doi: 10.3389/fpsyg.2026.1802059 (PMC13671976; doi:10.3389/fpsyg.2026.1802059)
Supplement: Supplementary file 1 [file Supplementary_file_1.docx]

Supplementary Material

# 1 Supplementary Data

**Supplementary Data 1: Semi-structured Interview Protocol**

**Introduction and Purpose:** This interview protocol was designed to explore the organisational adaptation experiences, knowledge transfer mechanisms, and institutional logic conflicts of repatriated engineers who returned to the Case Company (CC) following their postgraduate studies abroad. The questions specifically target the transition from global education to local organizational reality.

**Section 1: Demographic and Educational Background**

What is your age?

How many years have you been employed at this institution?

At which university did you complete your undergraduate degree?

In which university and country did you complete your postgraduate (Master’s) studies through the institutional scholarship?

**Section 2: Motivations for Migration and Repatriation**

5. What were your primary motivations for utilizing the institutional scholarship provided by this organization?

6. Why did you choose that specific university for your postgraduate studies abroad?

7. What factors influenced your decision to return to Turkey following the completion of your studies? (Exploring “push and pull” factors).

**Section 3: Organizational Support and Socialisation**

8. What kind of systemic or institutional support did you receive from the organization before your departure, during your studies abroad, and upon your return? (Assessing orientation and mentorship).

9. How did you feel on your first day of work upon repatriation? What were your initial expectations, and what were the actual conditions you encountered? (Identifying “reality shock” and “expectation gaps”).

**Section 4: Organisational Adaptation and Professional Satisfaction**

10. Do you believe that the specialized education you received abroad is sufficient for fulfilling the specific tasks and roles assigned to you within the institution? Why? (Exploring “expertise-role mismatch”).

11. What are your long-term career intentions once your compulsory service contract expires? (Assessing organizational commitment and retention).

**Section 5: Practical Recommendations and Redesign**

12. If you had unlimited resources and authority, how would you redesign the institution’s scholarship and repatriation system to optimize the adaptation of returning professionals?

**Supplemantary Table and Figures**

**Supplementary Table 1.** Participants Profiles

| **Case** | **Age** | **Education** | **Job Title** | **Experience in CC** | **Location in the CC** | **Duration of Interviews** |
| --- | --- | --- | --- | --- | --- | --- |
| M1 | 31 | MS | Junior Engineer | 3 years | Regional Directorate | 45 |
| M2 | 38 | Phd | Senior Engineer | 12 years | Headquarter | 75 |
| M3 | 35 | MS | Mid-level Engineer | 4.5 years | Regional Directorate | 49 |
| M4 | 37 | MS | Senior Engineer | 8 years | Regional Directorate | 54 |
| M5 | 34 | MS | Senior Engineer | 7 years | Headquarter | 58 |
| M6 | 38 | Phd | Senior Engineer | 12 years | Headquarter | 62 |
| M7 | 36 | MS | Senior Engineer | 11.5 years | Headquarter | 70 |
| M8 | 38 | MS | Senior Engineer | 10 years | Regional Directorate | 67 |
| M9 | 40 | MS | Senior Engineer | 10 years | Regional Directorate | 62 |
| M10 | 35 | MS | Senior Engineer | 8 years | Headquarter | 60 |
| M11 | 36 | MS | Senior Engineer | 7 years | Headquarter | 71 |
| M12 | 36 | MS | Mid-level Engineer | 5 years | Headquarter | 48 |

**Supplementary Figure 1.** Category of Cultural Enrichment

**Supplementary Figure 1. Category of Cultural Enrichment.** This figure presents the ‘‘Cultural Enrichment’’ code, which drew statements from a single participant regarding work-culture experiences acquired abroad.

**Supplementary Figure 2.** Category of Economic Stability

**Supplementary Figure 2. Category of Economic Stability.** This figure presents the ‘‘Economic Stability’’ code, in which a single participant linked sectoral uncertainty to pandemic-era oil-price volatility.
